# Values of MRI Imaging Presentations in the Hepatobiliary Phase, DWI and T2WI Sequences in Predicting Pathological Grades of Intrahepatic Mass-Forming Cholangiocarcinoma

**DOI:** 10.3389/fonc.2022.867702

**Published:** 2022-06-07

**Authors:** Li-Hong Xing, Li-Yong Zhuo, Jia-Ning Wang, Yan Zhang, Feng-Ying Zhu, Chu Wang, Xiao-Ping Yin, Bu-Lang Gao

**Affiliations:** ^1^ Affiliated Hospital of Hebei University/ School of Clinical Medicine of Hebei University, Baoding, China; ^2^ Computed Tomography (CT)/Magnetic Resonance Imaging (MRI) Room, Affiliated Hospital of Hebei University, Baoding, China; ^3^ Department of Radiology, Beijing YouAn Hospital, Capital Medical University, Beijing, China; ^4^ Eye Hospital and School of Ophthalmology and Optometry, Wenzhou Medical University, Wenzhou, China

**Keywords:** intrahepatic mass-forming cholangiocarcinoma, hepatobiliary phase, target sign, marginal low signal ring, pathological grade

## Abstract

**Objective:**

To retrospectively investigate the value of various MRI image menifestations in the hepatobiliary phase (HBP), DWI and T2WI sequences in predicting the pathological grades of intrahepatic mass-forming cholangiocarcinoma (IMCC).

**Materials and Methods:**

Forty-three patients of IMCCs confirmed by pathology were enrolled including 25 cases in well- or moderately-differentiated group and 18 cases in poorly-differentiated group. All patients underwent DWI, T2WI and HBP scan. The Chi square test was used to compare the differences in the general information. Logistic regression analysis was used to analyze the risk factors in predicting the pathological grade of IMCCs.

**Results:**

The maximal diameter of the IMCC lesion was < 3 cm in 11 patients, between 3 cm and 6 cm in 15, and > 6 cm in 17. Sixteen cases had intrahepatic metastasis, including 5 in the well- or moderately-differentiated group and 11 in the poorly-differentiated group. Seventeen (39.5%) patients presented with target signs in the DWI sequence, including 9 in the well- or moderately-differentiated group and 8 in the poorly-differentiated group. Twenty (46.5%) patients presented with target signs in the T2WI sequence, including 8 in the well- or moderately-differentiated group and 12 in the poorly-differentiated group. Nineteen cases (54.3%) had a complete hypointense signal ring, including 13 in the well- or moderately-differentiated group and 6 in the poorly-differentiated group. Sixteen (45.7%) cases had an incomplete hypointense signal ring, including 5 in the well- or moderately-differentiated group and 11 in the poorly-differentiated group. The lesion size, intrahepatic metastasis, T2WI signal, and integrity of a hypointense signal ring in HBP were statistically significantly different between two gourps. T2WI signal, presence or non-presence of intrahepatic metastasis, and integrity of hypointense signal ring were the independent influencing factors for pathological grade of IMCC.

**Conclusion:**

Target sign in T2WI sequence, presence of intrahepatic metastasis and an incomplete hypointense-signal ring in HBP are more likely to be present in poorly-differentiated IMCCs.

## Introduction

Cholangiocarcinoma is rare, including a group of rare highly aggressive hepatobiliary malignant tumors like ampulla of vater cancer, extrahepatic cholangiocarcinoma, gallbladder cancer, and intrahepatic cholangiocarcinoma (1). Intrahepatic cholangiocarcinoma (ICC) which arises from bile ductules to the second-order bile ducts is the second most common type of liver cancer after hepatocellular carcinoma (HCC), accounting for nearly 10-20% of all cholangiocarcinomas. ICCs generally have no obvious clinical symptoms and are mostly found in the elderly and in late stage. A great percentage of patients with Cholangiocarcinoma are thus unresectable, and curative surgical treatment is possible only in 25% of the patients, with a high recurrence rate even after radical surgical resection (2). Intrahepatic mass-forming cholangiocarcinoma (IMCC) is the most common subtype of ICC, accounting for about 60% of ICCs (3). About 90% of the ICC pathological types are adenocarcinoma, which can be classified into well-differentiated, moderately-differentiated and poorly-differentiated according to morphology. Poorly-differentiated ICC is associated with a shorter overall survival (4). Early radical surgical resection is the best treatment, with a 5-year survival rate of 5%-40% (5). Yussoff et al. (6) believed that effective surgical resection was related to improving the long-term survival rate for patients with early and well-differentiated ICC with a clear boundary.

IMCC can show a target sign in the magnetic resonance imaging (MRI) diffusion-weighted imaging (DWI) sequence, with hyperintense signal in the periphery but hypointense signal in the center on high b-value image (7). However, the incidence of target sign was different, with approximately 68.5%, 75% and 83.5% being reported in the literature (8–10), which may be caused by varied degrees of differentiation of the tumor. With decrease of the IMCC pathological grade, the lesion peripheral ADC (apparent diffusion coefficient) value also decreased (10), possibly increasing the presence of the target sign on DWI, however, a correlation between DWI signal characteristics and IMCC pathological grade has been rarely reported. The target sign may also appear on T2-weighted imaging (T2WI) of IMCC under the influence of the internal components of the IMCC lesions. When the lesion center is of dense fiber, coagulative necrosis or liquefied necrosis, T2WI image shows a relatively hyperintense signal in the periphery or a relatively hyperintense signal in the center to form a target sign. But, the correlation between the T2WI target sign and IMCC pathological grade is still unclear and needs further study.

Hepatocyte specific contrast agents have been widely used in the imaging of liver diseases. In using Gadobenate dimeglumine (Gd-BOPTA) as a contrast agent for MRI scanning of IMCCs, Mamone et al. (9) found that 28 out of 29 IMCCs presented with an uneven central hyperintense signal as a target sign or a cloud sign in the hepatobiliary phase (HBP). When using gadolinium ethoxybenzyl diethylenetriamine pentaacetic acid (Gd-EOB-DTPA) to differentiate IMCC from HCC on MRI, Kim et al. (8) found that IMCC lesions showed three signals in HBP: uniform hypointense signal, mixed hypointense signal or Mosaic sign, and a target sign or multilayer target sign, among which the target sign or multilayer target sign was the most common. At present, most studies focused on distinguishing IMCC from HCC or atypical liver abscess by using the target sign in HBP (11–13). The correlation of IMCC characteristics and enhancement with patients’ prognoses, lymph node metastasis and microvascular invasion (MVI) was also a focus of research (14, 15). However, few studies investigated the value of MRI imaging presents in HBP, T2WI and DWI in predicting the pathological grade of IMCCs. Mamone et al. (9) found that the continuous hypointense signal ring at the edge of lesions in HBP had developed from a discontinuous hypointense signal ring in the delayed stage of IMCC. Nonetheless, they didn’t analyze the existence and integrity of a hypointense signal ring at the edge of lesions in HBP, nor did they explore the relationship of the hypointense signal ring with pathological grade of IMCC. Consequently, this study was performed to investigate the value of MRI DWI, T2WI and HBP imaging features using the Gd-BOPTA as a contrast agent in predicting the IMCC pathological grade before surgical treatment.

## Materials and Methods

### Subjects

This retrospective cross-sectional one-center study was performed from October 2017 to December 2021 with the approval of the ethics committee of our hospital, and all patients had provided written informed consent to participate. Inclusion criteria were patients with ICC confirmed by surgery or pathology, unclear pathological grade at pathology report before surgery, MRI plain and enhanced scan before pathological staining, and no treatment before pathological examination. Exclusion criteria were patients with pathologically-confirmed hepatocellular carcinoma, other tumors or poor image quality for correct analysis.

### Image Acquisition

MRI was performed using the GE Discovery MR 750 3.0T (GE Discovery MR 750 3.0T, GE Healthcare, Milwaukee, WI, USA) superconducting scanning device with the abdominal phase controlled front coil. Axial fat-suppression T2WI, axial DWI, axial VIBE mask, axial VIBE three phase dynamic enhanced scan and hepatobiliary phase scan were performed. The contrast agent was Gd-BOPTA (Multihance, Bracco Imaging, Milano, Italy) *via* bolus injection through the elbow vein, with a dose of 0.1 mmol/kg and an injection flow rate of 2mL/s, and a wash with at least 20 mL physiological saline was performed at the same rate after injection of the contrast agent. Scanning at the early and late arterial phases, portal phase, and equilibrium phase was performed at 15–20 s, 35–40 s, 60 s and 180 s, respectively, after contrast injection. The HBP scanning was conducted 90-120 minutes later.

### Pathological Examination

All postoperative specimens were sent for pathological staining. For cases with poorly-differentiated IMCC and difficult staining to determine the tumor histological type, immunohistochemical staining was further performed to confirm the diagnosis. The pathological grade of IMCCs was divided into well-differentiated, moderately-differentiated and poorly-differentiated.

### Image Analysis and Pathological Evaluation

The images were assessed by two deputy chief physicians of imaging diagnosis without knowing the pathology. If there was any disagreement, an agreement was reached through negotiation. A target sign was defined as peripheral relatively hyperintense signal or central relatively hyperintense signal, whereas a multilayer target sign as a peripheral hypointense signal, a hyperintense signal in the middle layer, and a hypointense signal in the center. MRI findings were described according to the following parameters: lesion size (maximal diameter < 3 cm, between 3 cm and 6 cm, and > 6 cm), DWI signal (target sign and non-target sign), T2WI signal (target sign and non-target sign), HBP signal (target sign, multilayer target sign, hypointense signal, and equal to hyperintense signal), presence and integrity of a hypointense signal ring at the edge of lesion in HBP (presence of a complete ring, presence of an incomplete ring, or non-presence of a ring), presence or non-presence of capsule shrinkage, lymph node metastasis, tumor emboli, bile duct dilation, abnormal perfusion, and intrahepatic metastasis.

### Statistical Analysis

The SPSS 22.0 statistical software (IBM, Chicago, IL, USA) was used for statistical analysis. Measured data were presented as mean ± standard deviation if in normal distribution and tested with the paired t test. Enumeration data were presented as frequency and percentage and tested with the Chi square test. Univariate logistic regression analysis was performed to analyze the risk factors of the IMCC differentiation degree. For factors with significant statistical differences in univariate analysis, multivariate logistic regression analysis was used to screen independent risk factors related to the degree of IMCC differentiation, and the odds ratio (OR) and 95%CI were calculated. p<0.05 was considered statistically significant.

## Result

### Clinical and Pathological Information

**Figure 1:**
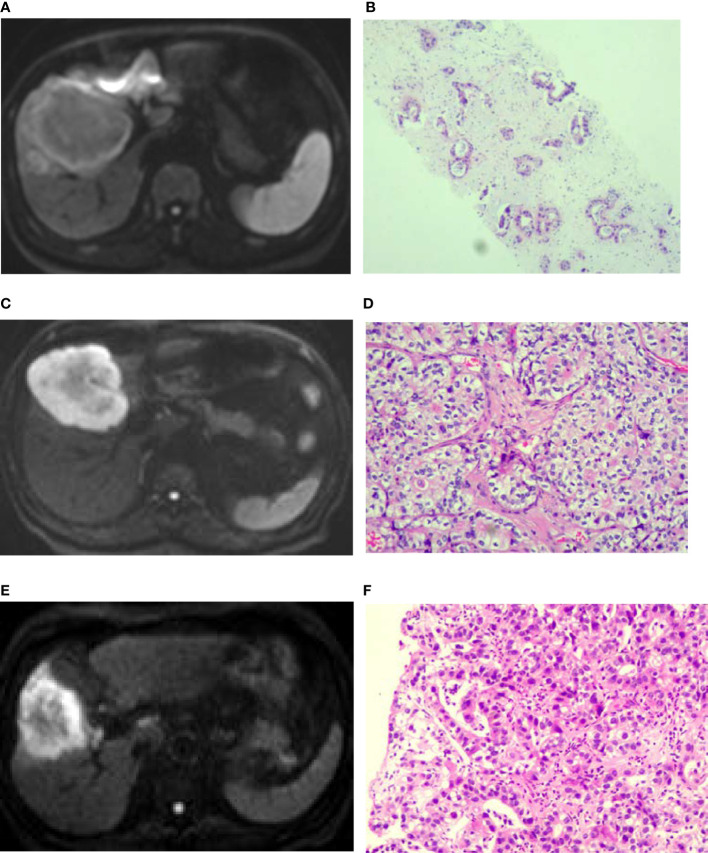
Diffusion weighted imaging (DWI) of intrahepatic mass-forming cholangiocarcinomas (IMCCs) with different differentiations. **(A, B)** A target sign was shown in a well-differentiated IMCC lesion on DWI with peripherally relatively-hyperintense signal. **(C, D)** A target sign was shown in a moderately-differentiated IMCC lesion on DWI with peripherally relatively-hyperintense signal. **(E, F)** A target sign was shown in a poorly-differentiated IMCC lesion on DWI with peripherally relatively-hyperintense signal. Pathological staining: ×200.

**Figure 2:**
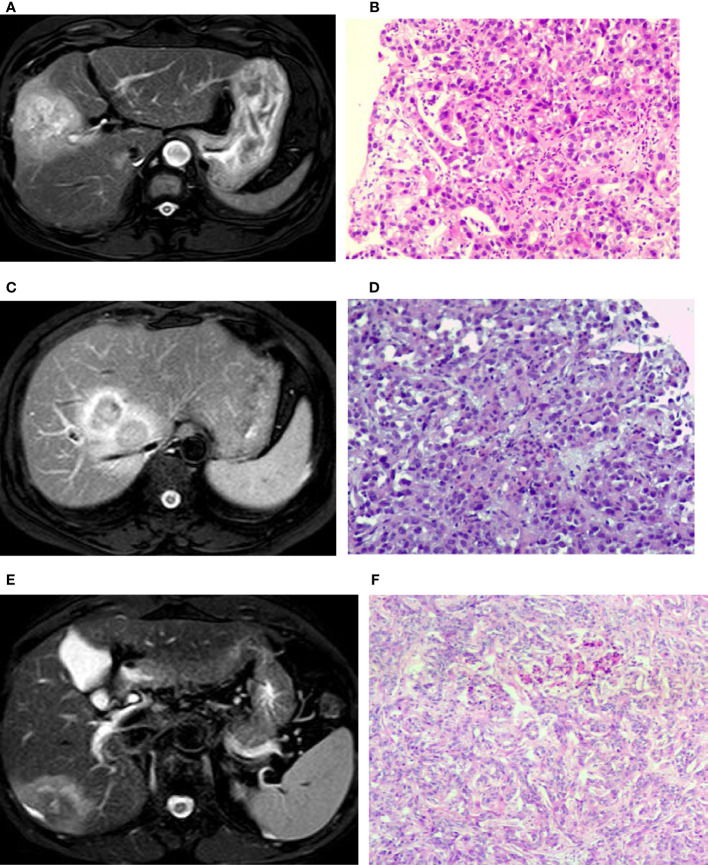
T2 weighted imaging (T2WI) of intrahepatic mass-forming cholangiocarcinomas (IMCCs) with different differentiations. **(A, B)** A target sign was shown in a poorly-differentiated IMCC lesion on T2WI image with centrally relatively-hyperintense signal. **(C, D)** A target sign was shown in a poorly-differentiated IMCC lesion on T2WI image with peripherally relatively-hyperintense signal. **(E, F)** A target sign was shown in a moderately-differentiated IMCC lesion on T2WI with peripherally relatively hyperintense signal. Pathological staining: ×200.

**Figure 3:**
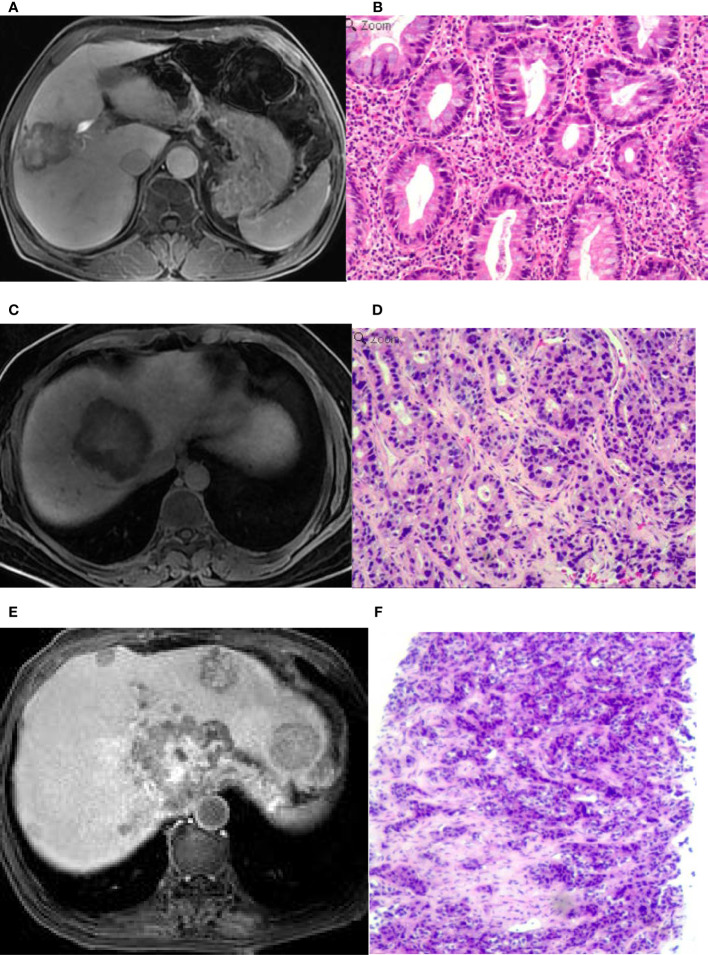
An intrahepatic mass-forming cholangiocarcinoma (IMCC) lesion on magnetic resonance imaging (MRI) in the hepatobiliary phase (HBP) and in pathological staining. **(A)** A target sign was shown on the HBP image with an incomplete hypointense signal ring at the lesion edge. **(B)** Pathological staining showed that the IMCC lesion was poorly differentiated. **(C)** A target sign was shown on the HBP image, with a complete hypointense signal ring at the lesion edge. **(D)** Pathological staining demonstrated that the IMCC lesion was moderately-differentiated. **(E)** A target sign was shown on the HBP image with an incomplete hypointense signal ring at the lesion edge, and multiple metastases were seen in the liver, which also presented as a target sign. **(F)** Pathological staining showed that the IMCC lesion was poorly differentiated. Pathological staining: ×200.

**Figure 4:**
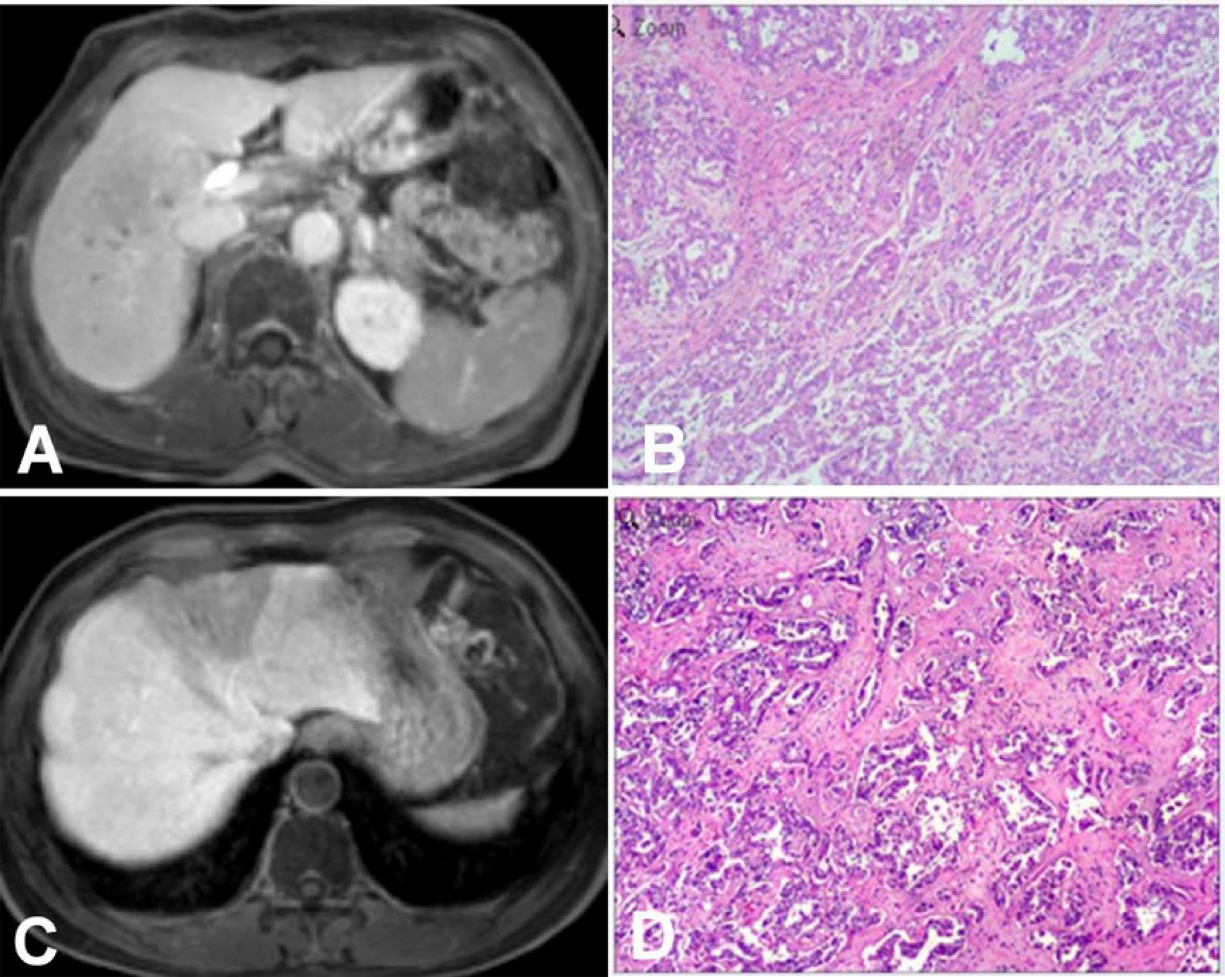
Intrahepatic mass-forming cholangiocarcinomas (IMCCs) on magnetic resonance imaging (MRI) in the hepatobiliary phase (HBP). **(A, B)** An IMCC lesion was demonstrated as hypointense signal intensity on HBP MRI with a blurred boundary but no hypointense signal ring at the lesion edge **(A)**, and pathological staining showed that the IMCC was moderately differentiated **(B)**. **(C, D)** An IMCC lesion was demonstrated as hypointense signal intensity on HBP MRI with a clear boundary but no hypointense signal ring at the lesion edge **(C)**, and pathological staining showed that the IMCC was well differentiated **(D)**. Pathological staining: ×200.

Forty-three patients were enrolled, including 27 males (62.8%) and 16 females (37.2%) aged 60.98 ± 11.02 years. The lesion was pathologically confirmed by surgery or needle biopsy at final report in all patients. Seven patients had a history of chronic viral hepatitis B and one patient had a history of cirrhosis. According to the degree of pathological differentiation, the patients were divided into two groups according to the tumor differentiation: well- or moderately-differentiated group (n=25), and poorly-differentiated group (n=18) (**Table 1** and **Figures 1**–**4**).

**Table 1:** Clinical and pathological information.

Clinical characteristics		*n*	%
Gender	male	27	62.8
	female	16	37.2
Pathological grade	Poorly differentiated	18	41.9
	Well- or moderately- differentiated	25	58.1
Lesion size (cm)	<3	11	25.6
	3-6	15	34.9
	>6	17	39.5
T2WI signal	no-target sign	23	53.5
	target sign	20	46.5
DWI signal	no-target sign	26	60.5
	target sign	17	39.5
HBP signal	hypointense signal	8	18.6
	target/multi target sign	35	81.4
Presence of hypointense signal ring in HBP integrity of hypointense signal ring in HBP	presence non-presence complete incomplete	35 8 19 16	81.4 18.6 54.3 45.7

DWI, magnetic resonance imaging diffusion weighted imaging; T2WI, T2 weighted imaging; HBP, hepatobiliary phase.

### General Imaging in IMCCs With Different Pathological Grades

The maximal diameter of the IMCC lesion was < 3 cm in 11 patients, between 3 cm and 6 cm in 15, and > 6 cm in 17. Sixteen cases had intrahepatic metastasis, including 5 in the well- or moderately-differentiated group and 11 in the poorly-differentiated group (**Figures 1**–**4**). Capsule shrinkage was present in four patients in the well- or moderately-differentiated group and in five patients in the poorly-differentiated group. Lymph node metastasis was present in two patients in the well- or moderately-differentiated group and in four patients in the poorly-differentiated group were 4 patients. Tumor emboli were present in three patients in the well- or moderately-differentiated group and in four patients in the poorly-differentiated group. Abnormal perfusion occurred in ten patients in the well- or moderately-differentiated group and in ten patients in the poorly-differentiated group. Bile duct expansion was present in eight patients in the well- or moderately-differentiated group and in eleven patients in the poorly-differentiated group. Lesion size (*χ* ^2^=7.528, p=0.023) and intrahepatic metastasis (*χ* ^2^=5.570, p=0.006) were statistically significantly different in the univariate logistic regression analysis between the two gourps (**Tables 1**, **2**).

**Table 2:** Univariate analysis results of pathological grade [n(%)].

factors		pathological grade		χ^2^	*P*
		Poorly differentiated (n = 18)	Well- or moderately-differentiated (n = 25)		
Lesion size (cm)	<3	1 (5.6)	10 (40.0)	7.528	0.023
	3~	8 (44.4)	7 (28.0)		
	>6	9 (50.0)	8 (32.0)		
T2WI signal	no-target sign	6 (33.3)	17 (68.0)	5.055	0.025
	target sign	12 (66.7)	8 (32.0)		
DWI signal	no-target sign	10(55.6)	16 (64.0)	0.312	0.576
	target sign	8 (44.4)	9 (36.0)		
HBP signal	hypointense signal	1 (5.6)	7 (28.0)	2.157	0.142
	target sign	17 (94.4)	18 (72.0)		
presence of hypointense signal ring in HBP	presence	17 (94.4)	18 (72.0)	2.157	0.142
	non-presence	1 (5.6)	7 (28.0)		
integrity of hypointense signal ring in HBP	complete	6 (35.3)	13 (72.2)	4.804	0.028
	incomplete	11 (65.7)	5(27.8)		
capsule shrinkage	non-presence	13 (72.2)	21 (84.0)	0.310	0.578
	presence	5 (27.8)	4 (16.0)		
lymph node metastasis	non-presence	14 (77.8)	23 (92.0)	0.777	0.378
	presence	4 (22.2)	2 (8.0)		
tumor emboli	non-presence	14 (77.8)	22 (88.0)	0.228	0.633
	presence	4 (22.2)	3 (12.0)		
bile duct dilation	non-presence	7 (38.9)	17 (68.0)	3.596	0.058
	presence	11 (61.1)	8 (32.0)		
abnormal perfusion	non-presence	8 (44.4)	15 (60.0)	1.018	0.313
	presence	10 (55.6)	10 (40.0)		
intrahepatic metastasis	non-presence	7 (38.9)	20 (80.0)	5.570	0.006
	presence	11 (61.1)	5 (20.0)		

DWI, magnetic resonance imaging diffusion weighted imaging; T2WI, T2 weighted imaging; HBP, hepatobiliary phase.

### DWI, T2WI and HBP Signal in IMCCs With Different Pathological Grades

Seventeen (39.5%) patients presented with target signs in the DWI sequence, including 9 in the well- or moderately-differentiated group (**Figures 1A, B**) and 8 in the poorly-differentiated group (**Figure 1C**). Twenty (46.5%) patients presented with target signs in the T2WI sequence, including 8 in the well- or moderately-differentiated group (**Figure 2C**) and 12 in the poorly-differentiated group (**Figures 2A, B**). Thirty-five (81.4%) patients presented with a target or multilayer target sign in HBP, including 18 in the well- or moderately-differentiated group (**Figures 3C, D**) and 17 in the poorly-differentiated group (**Figures 3A, B, E, F**). Eight patients (18.6%) had hypointense signal in HBP(**Figures 4A–D**). Thirty-five cases (81.4%) had a hypointense signal ring at the lesion edge in HBP, including 18 in the well- or moderately-differentiated group and 17 in the poorly-differentiated group. Nineteen cases (54.3%) had a complete hypointense signal ring, including 13 in the well- or moderately-differentiated group (**Figures 3C, D**) and 6 in the poorly-differentiated group. Sixteen (45.7%) cases had an incomplete hypointense signal ring, including 5 in the well- or moderately-differentiated group and 11 in the poorly-differentiated group (**Figures 3A, B, E, F**). The T2WI signal (*χ* ^2^=5.055, p=0.025) and integrity of a hypointense signal ring (*χ* ^2^=4.804, p=0.028) at the edge of lesion in HBP were statistically significantly different in the univariate logistic regression analysis between the two groups (**Tables 1**, **2**).

### Multivariate Logistic Regression Analysis

Lesion size, T2WI signal, integrity of hepatobiliary peripheral hypointense signal ring in HBP, and presence or non-presence of intrahepatic metastasis which were significant in univariate logistic regression analysis were entered into the multivariate logistic regression analysis. The result showed that T2WI signal, presence or non-presence of intrahepatic metastasis, and integrity of hypointense signal ring were the independent influencing factors for pathological grade of IMCC. The IMCCs with the T2WI target sign, presence of intrahepatic metastasis and an incomplete hypointense signal ring in HBP were more likely to be pathological poorly differentiated (**Tables 3**, **4**).

**Table 3:** Factor assignment.

variable	assignment
T2WI signal	no-target sign=1 target sign=2
presence or non-presence intrahepatic metastasis	non-presence =1 presence=2
integrity of hypointense signal ring in HBP	complete=1 incomplete=2
pathological grade	well and moderately differentiated=0 poorly differentiated=1

**Table 4:** Multivariate logistic regression analysis of independent risk factors for pathological grade.

variable	b	S_b_	Wald-*χ* ^2^	*P*	OR(95CI)
T2WI singnal	3.142	1.361	5.329	0.021	23.15 (1.61-333.38)
presence or non-presence intrahepatic metastasis	2.124	1.012	4.406	0.036	8.36 (1.15-60.74)
integrity of hypointense signal ring in HBP	2.616	1.254	4.352	0.037	13.68 (1.17-159.67)
constant	-12.022	4.555	6.966	0.008	–

T2WI, T2 weighted imaging of magnetic resonance imaging; HBP, hepatobiliary phase.

## Discussion

In this study investigating the predictive value of various MRI image menifestations for the pathological grade Of IMCCs, it was found that T2WI signal, intrahepatic metastasis, integrity of hypointense-signal ring were independent influencing factors for predicting the pathological grade of IMCCs. There were no statistically significant differences in the target sign of DWI and HBP between the well- or moderately-differentiated group and poorly-differentiated group.

In the study, the rate of target sign in HBP of IMCC was 81.4%. The pathological basis of the target sign is that the center is rich in fibrous tissue, which can accumulate the contrast agent to present with a hyperintense signal. In the peripheral area of the lesion, tumor cells are abundant while fibrous tissues are fewer, thus resulting in a relatively hypointense signal in the periphery (8, 9, 13, 16). However, the target sign is not unique to IMCCs in HBP. Approximately 65.0% of scirrhous HCC show a target appearance in HBP, with accumulation of contrast agent in the center (17). In addition, many intrahepatic metastatic adenocarcinomas also present with a target sign in HBP (9 **)**. When the metastases are isolated and the tumor history is unclear, it is difficult to distinguish them from IMCCs. The target sign in HBP was not significantly different in the well- or moderately-differentiated group as compared with the poorly differentiated group, however, the integrity of marginal hypointense signal ring in HBP was an independent risk factor for poorly differentiated IMCCs This indicates that an incomplete marginal hypointense signal ring is more common in poorly differentiated IMCC lesions. One possible reason for this is that in IMCC lesions with poor differentiation, the ADC value is decreased in the periphery (10), and the extracellular space is smaller. This may cause imbalanced distribution and growth of tumor cells (18), with too few tumor cells or thin distribution of tumor cells in some areas and subsequently a discontinued and incomplete hypointense signal ring. On the other hand, in IMCCs with poor differentiation, the lesion tends to be non-spherical or irregular (14), and the edge hypointense signal ring tends to be uneven in thickness and poorly displayed, causing visual interruption and discontinuity. Moreover, compared with well-differentiated IMCC lesions, a poorly-differentiated IMCC lesion is more likely to have cystic and necrosis in the center which may present with a hypointense signal (13). The range of hypointense signal in the center is larger and irregular while the range of hypointense signal in the periphery is relatively smaller and irregular, which may lead to unclear display of the local hypointense signal ring in some area.

In this study, the target sign in T2WI sequence and intrahepatic metastases may also be used to predict the pathological grade of IMCC. In poorly-differentiated tumors, tumor cells grow very fast, and the central part of the tumor will be more prone to cystic degeneration and necrosis due to insufficient blood supply (13). Thus, a higher signal may be shown in the center rather than in the periphery in T2WI sequence, forming the target sign. Tumor grade is one of the risk factors for microvascular invasion (14). With decrease in the tumor differentiation, microvascular invasion will likely occur (14), and tumor cells will likely metastasize through blood vessels, in or out of the liver.

The current study showed that the target sign in the DWI sequence between the well- or moderately-differentiated group and poorly differentiated group was not statistically significant. This may be related to the ADC value of the lesion because the ADC value decreases with decrease of the differentiation degree of the tumor (19). The mean ADC value of poorly-differentiated tumors is significantly lower than that of well- or moderately-differentiated tumors (20). Although the ADC value reflects the richness of tumor cells and pathological grade (18), the peripheral hyperintense signal in DWI sequence of the IMCC lesion is not only related to the richness of tumor cells, but also affected by a variety of factors such as T2 shine-through effects, T2 blackout effects, b value, and field intensity (13, 21). The term “T2-shine-through” has been coined to describe the substantial contribution of T2 hyperinstensity observed on DWI (21). The correlation we observed between the T2-hypointensity and DWI suggested a contribution of the “T2 blackout effect” (21). When the center of the IMCC lesion is composed of dense collagen or coagular necrosis, the T2WI signal is decreased and may be directly transformed into a dark central area on DWI (13), resulting in the presence of the target sign. When the center of the IMCC lesion is of liquefaction and necrosis, the signal is obviously hyperintense on T2WI, and the T2 penetrating effect will also be transformed into the central hyperintense signal area on DWI, leading to the presence of the target sign. Therefore, the target sign in the DWI sequence cannot accurately predict the pathological grade of IMCC.

In the current study, the pathological type of all the IMCC lesions is adenocarcinomas. Adenocarcinoma is the most common pathological type of ICCs, accounting for 90% (4), with a ductal, tubular or cord-like pattern, and with variable, and often abundant, fibrous stroma (1). Rare variants of ICC include squamous or adenosquamous carcinoma, lymphoepithelioma-like carcinoma related to Epstein-Barr virus infection, and sarcomatous carcinoma (1). Histologically, ICCs are usually well- to moderately- differentiated adenocarcinomas, and are recognized as two histological subtypes: large and small duct types. The large duct type, originating in the large intrahepatic biliary ducts near the hepatic hilus, is most frequently seen as moderately-differentiated tumor. The small duct type, which mostly occurs in the hepatic periphery, is primarily seen as well-differentiated tumor (1). It is worth noting that the histological subtype reflects the high molecular heterogeneity of iCCAs and can be ascribed to different cells of origin and pathogenesis.

There were some limitations in the current study, including a small sample size, enrollment of Chinese patients only, one center study design, non-randomization, and retrospective study design, which may all affect the outcomes of the study and produce some bias. Furthermore, more images of T1WI, T1 dynamic enhancement and differential diagnosis of IMCC from HCC or intrahepatic metastases should be included to describe various parameters and different aspects of lesions in correlation with the histopathology. All these issues have to be resolved in future studies for achieving better outcomes.

In conclusion, T2WI signal, presence of intrahepatic metastasis, integrity of a hypointense signal ring are the independent risk factors for predicting the pathological grade of IMCCs. Tagert sign in T2WI sequence, presence of intrahepatic metastasis, and an incomplete hypointense signal ring in HBP are more likely to suggest a pathologically poorly differentiated tumor. The target sign in DWI sequence and HBP are not the risk factors for predicting the pathological grade of IMCCs.

## Data Availability Statement

The original contributions presented in the study are included in the article/supplementary material. Further inquiries can be directed to the corresponding author.

## Ethics Statement

The studies involving human participants were reviewed and approved by Ethics committee of Affiliated Hospital of Hebei University. The patients/participants provided their written informed consent to participate in this study.

## Author Contributions

Conception and design of the research, L-HX; Acquisition of data, L-HX, YZ, and F-YZ; Analysis and interpretation of the data, L-HX and J-NW; Statistical analysis, L-HX; Obtaining financing, X-PY and L-HX; Writing of the manuscript, L-HX and B-LG; Critical revision of the manuscript for intellectual content, XPY, CW, and B-LQ; All authors contributed to the article and approved the submitted version.

## Funding

The Outstanding Young Scientific Research and Innovation Team of Hebei University (605020521007), Natural Science Foundation of Hebei (H2021201017), CT Radiomics Study on the Correlation between Colorectal Liver Metastasis and Microsatellite Instability, Medical Discipline Cultivation Project (2020B05), CT Radiomics Study on the Correlation between Colorectal Liver Metastasis and Microsatellite Instability, The Financial Department of Hebei Province, Health commission of Hebei Province (361007), CT Radiomics Study on the Correlation between Colorectal Liver Metastasis and Microsatellite Instability, Key Science and Technology Research Program of Hebei Province (20210789), the study of IVIM-DWI combined with Gd-BOPTA in hepatobiliary stage in the identification of Intrahepatic cholangiocarcinoma and atypical liver abscess, Baoding Science and Technology Bureau project (2041ZF176), study on the differential value of IVIM-DWI combined with Gd-BOPTA in hepatobiliary stage for intrahepatic mass-forming cholangiocarcinoma and atypical liver abscess.

## Conflict of Interest

The authors declare that the research was conducted in the absence of any commercial or financial relationships that could be construed as a potential conflict of interest.

## Publisher’s Note

All claims expressed in this article are solely those of the authors and do not necessarily represent those of their affiliated organizations, or those of the publisher, the editors and the reviewers. Any product that may be evaluated in this article, or claim that may be made by its manufacturer, is not guaranteed or endorsed by the publisher.
